# Heterogeneous tumor‐immune microenvironments between primary and metastatic carcinoid tumors differentially respond to anti‐PD‐L1 antibody therapy

**DOI:** 10.1111/1759-7714.13772

**Published:** 2020-12-09

**Authors:** Shinya Sakata, Kosuke Imamura, Yuka Tajima, Yuiko Masuda, Ryo Sato, Chieko Yoshida, Shinichiro Okamoto, Sho Saeki, Yusuke Tomita, Takuro Sakagami

**Affiliations:** ^1^ Department of Respiratory Medicine Kumamoto University Hospital Kumamoto Japan; ^2^ Department of Respiratory Medicine, Graduate School of Medical Sciences Kumamoto University Kumamoto Japan; ^3^ Laboratory of Stem Cell and Neuro‐Vascular Biology Genetics and Developmental Biology Center, National Heart, Lung, and Blood Institute, National Institutes of Health Bethesda Maryland USA

**Keywords:** Carcinoid, cytotoxic T lymphocyte antigen 4 (CTLA‐4), heterogeneity, immune checkpoint inhibitor (ICI), neuroendocrine tumors (NET)

## Abstract

A pulmonary carcinoid tumor is a rare tumor that lacks a validated therapeutic approach for unresectable disease. Understanding the intersite tumor‐immune heterogeneity is essential to harness the immune system for cancer therapy. However, little is known about the tumor‐immune microenvironment (TIME). Here, we describe a patient who had heterogeneous TIME between primary and metastatic carcinoid tumors which differentially responded to chemoimmunotherapy. A 72‐year‐old man was diagnosed with an advanced pulmonary carcinoid tumor. CT‐guided biopsies of lung and scapular tumors confirmed typical carcinoid (PD‐L1, 1%–24%) and atypical carcinoid tumors (PD‐L1, negative), respectively. Although the primary lung carcinoid tumor showed a partial response, the scapular tumor was significantly enlarged after two cycles of anti‐PD‐L1 antibody therapy in combination with carboplatin plus etoposide. We performed quantitative pathology imaging analysis with fluorescent multiplex immunohistochemistry. CD8^+^ T cell infiltration was detected in the PD‐L1‐positive primary lung tumor nest; however, it was mostly restrained in the stroma in a PD‐L1‐negative metastatic scapular tumor. Treg infiltrations into both tumor nests and stroma were detected in the lung tumor, which were not detected in the metastatic scapular tumor. This study provides the first evidence of coexistence of heterogeneous TIME within a single individual with a pulmonary carcinoid tumor. This study may provide new insights into the mechanism of primary resistance to chemoimmunotherapy in pulmonary carcinoid tumors.

## Graphical abstract

We describe a patient who had heterogeneous TIME between primary and metastatic carcinoid tumors which differentially responded to chemoimmunotherapy. CD8+ T cell infiltration was detected in PD‐L1‐positive primary lung tumor nest, however, which was mostly restrained in the stroma in a PD‐L1‐negative metastatic scapular tumor. This study may provide new insights into the mechanism of primary resistance to chemoimmunotherapy in pulmonary carcinoid tumors.

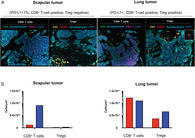

## Introduction

Typical and atypical carcinoids are classified as highly differentiated malignant neuroendocrine tumors (NET).^1^ Due to their rarity, pulmonary carcinoids lack a validated therapeutic approach for unresectable disease.^2^, ^3^ Therefore, development of new therapeutic options is urgently needed.

Although immune checkpoint inhibitors (ICIs) have revolutionized the treatment of non‐small cell lung cancer (NSCLC), their impact on small cell lung cancer, which is in the same spectrum with typical and atypical carcinoids, is limited.^4^, ^5^ Drug development of immunotherapy targeting pulmonary NET including carcinoids is ongoing.^1^, ^3^, ^6^ However, there is only very limited evidence for immunotherapy in patients with pulmonary carcinoids, with early trials suggesting a low‐level activity in patients treated with single agent therapy,^3^, ^6^ highlighting the importance of investigating the heterogeneity of tumor‐immune microenvironments (TIME) in carcinoids.

TIME have an influence on tumor initiation and response to therapy.^7^, ^8^, ^9^ Thus, understanding the intersite tumor‐immune heterogeneity is essential to harness the immune system for cancer therapy. However, little is known about TIME in carcinoid tumors.^10^ Here, we describe a patient who had heterogeneous TIME between primary and metastatic carcinoid tumors which differentially responded to chemoimmunotherapy. This study may provide new insights into the mechanism of primary and acquired resistance to immunotherapy in carcinoid tumors.

### Case report

**Figure 1:**
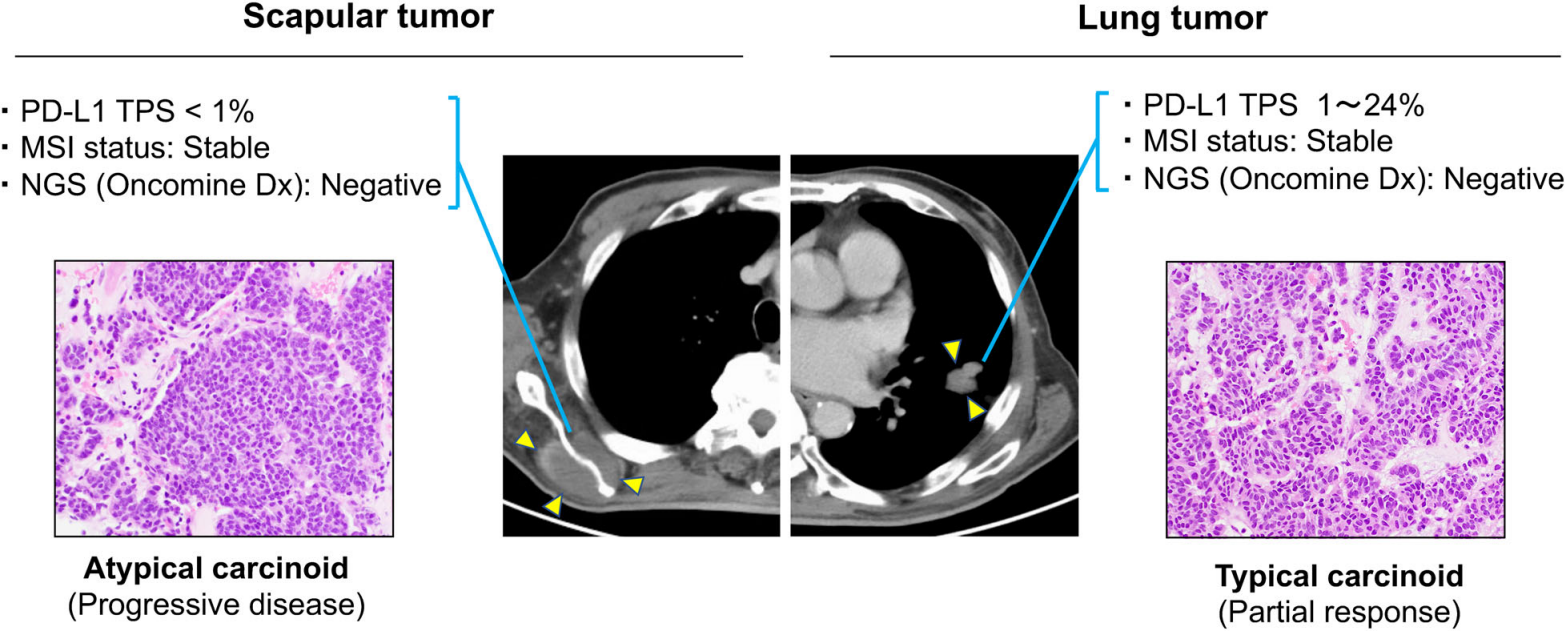
Key imaging and clinicopathological results. Chest computed tomography (CT) scan show masses in the left lower lung and right scapular (yellow arrows). Histopathological findings obtained from the left lower primary lung tumor show small to medium‐size round nuclei and lightly acidic cytoplasm distributed in a sheet‐like form. The mitotic count was less than 1 in 10/high power field (HPF). Immunohistochemical staining was positive for thyroid transcription factor‐1 (TTF‐1), synaptophysin and insulinoma‐associated 1 (INSM‐1). The Ki‐67 labeling index was 4%–5%. Based on these findings, the lung tumor was diagnosed as a typical pulmonary carcinoid tumor. The programmed death‐ligand 1 (PD‐L1) tumor proportion score (TPS) of the lung tumor was 1%–24% (clone 22C3, pharmDx kit). Next‐generation sequencing (NGS) (Oncomine Dx Target Test multi‐CDx system) and microsatellite instability (MSI) tests were negative. After two cycles of atezolizumab in combination with carboplatin plus etoposide, CT scan of the chest showed a partial response in the lung lesion according to Response Evaluation Criteria in Solid Tumor (RECIST) version 1.1. In the biopsy sample from the scapular tumor, the mitotic count of the scapular lesion was 2–3/HPF and diagnosed as an atypical carcinoid. The PD‐L1 TPS was negative (<1%). Oncomine Dx Target Test multi‐CDx system and MSI tests were negative. A post‐therapy CT scan showed progressive disease in the scapular lesion.

A 72‐year‐old man was diagnosed with advanced pulmonary carcinoid with multiple bone metastases (Fig 1). CT‐guided biopsies of lung and scapular tumors confirmed the diagnoses of typical carcinoid (PD‐L1, 1%–24%; Oncomine Dx target test, negative; microsatellite instability [MSI], negative) and atypical carcinoid (PD‐L1, negative; Oncomine Dx, negative; MSI, negative), respectively.

The patient received atezolizumab in combination with carboplatin plus etoposide as first‐line therapy. Although a CT scan showed a partial response in the primary lung lesion, the scapular tumor was significantly enlarged after two cycles of chemoimmunotherapy. There was no antitumor effect on other bone metastases similar to the scapula tumor. Thus, a progressive disease was evaluated according to RECIST version 1.1.

**Figure 2:**
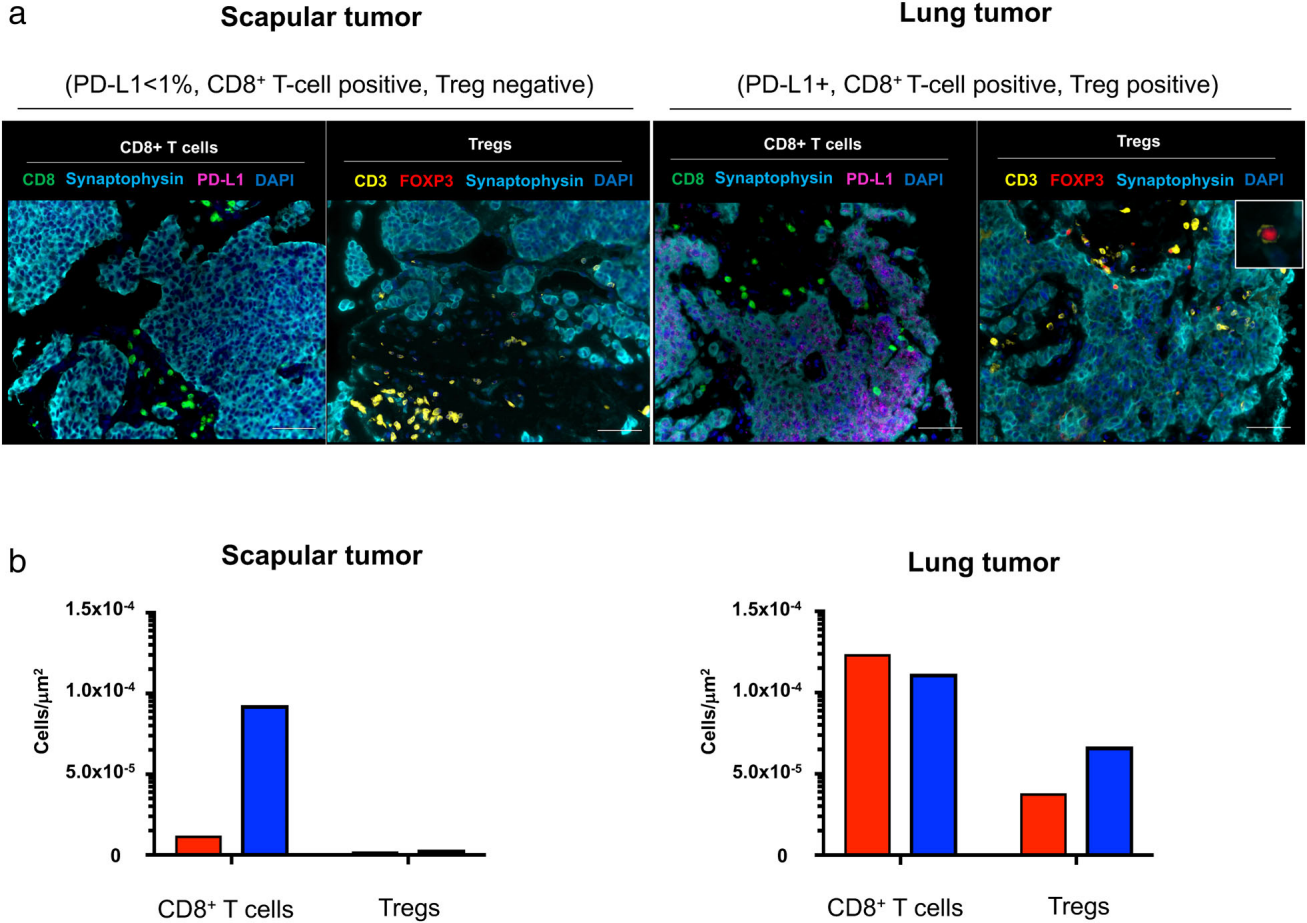
Multiplex fluorescent immunohistochemistry results. (**a)** Representative images of scapular and lung tissues are shown. Formalin‐fixed paraffin‐embedded sections of scapular and lung tumors were stained by one of two sequences of primary antibodies, PD‐L1 (purple), synaptophysin (light blue) and CD8 (green), or synaptophysin, FOXP3 (red) and CD3 (yellow) respectively. Nuclei were counterstained with DAPI (blue). In the scapular tumor, CD8^+^ T cells were distributed mainly in the tumor stroma, and CD3^+^FOXP3^+^ Tregs were scarcely observed. In lung tumor, CD8^+^ T cell infiltrations into PD‐L1‐positive tumor cell nest were observed, and Treg also infiltrated into the synaptophysin‐positive tumor cell nest. The insert panel in the right upper panel shows a Treg at high magnification. Scale bars, 50 μm are shown in each panel. (**b)** Quantification results of CD8^+^ T cells and CD3^+^FOXP3^+^ Tregs are shown. High‐speed scanning of whole slide images was performed on stained tissue sections. Images of full sections were acquired and analyzed with automated quantitative pathology imaging system. All images were analyzed and statistics of the number of CD8^+^ T cells and CD3^+^FOXP3^+^ Tregs were generated automatically. Cells were classified as tumor nest (red) or tumor stroma (blue) according to the relationship with synaptophysin‐positive tumor cells. Most CD8^+^ T cells in the scapular tumor were classified as tumor stroma, and few Tregs were detected in the scapular tumor. In the lung tumor, CD8^+^ T cells were distributed almost equally in the tumor nest and stroma. Tregs in lung tumor were counted more than in the scapular tumor. The detailed methods are described in Supplementary Text S1. (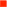) Tumor nest, (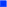) Tumor stroma

Understanding the intersite tumor‐immune heterogeneity is essential to harness the immune system for cancer therapy. However, little is known about TIME in pulmonary carcinoid tumors. To further explore this case, we investigated the immune contexture of pretreatment primary and metastatic tumors by fluorescent multiplex immunohistochemistry (Supplementary Text S1), which can capture multidimensional data related to tissue architecture, spatial distribution of multiple cell phenotypes, and co‐expression of signaling.^11^, ^12^ Synaptophysin and PD‐L1 were simultaneously stained and the tissue localizations of tumor‐infiltrating lymphocytes (TIL) were evaluated. CD8^+^ T cells and immunosuppressive CD3^+^FOXP3^+^ regulatory T cells (Tregs) in the tumor nest and surrounding stroma were profiled and quantified by quantitative pathology imaging system. CD8^+^ T cell infiltrations were detected in the tumor nest of PD‐L1‐positive primary lung carcinoid; however, they were mostly restrained in the stroma of the PD‐L1‐negative metastatic scapular tumor. Treg infiltrations into both tumor nests and stroma were detected in the primary lung carcinoid tumor, however, which were not detected in the metastatic scapular tumor (Fig 2).

**Figure 3:**
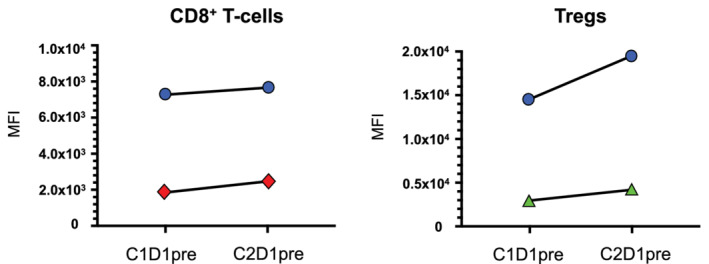
Peripheral blood immune subset analyses. Immune checkpoint receptors and Ki67 expressions of peripheral CD8^+^ T cells and Tregs in response to anti‐PD‐L1 antibody therapy in combination with chemotherapy are shown. Peripheral blood mononuclear cells (PBMCs) were obtained before therapy on cycle one day one (C1D1pre), and prior to treatment on C2D1(C2D1pre). Multiparameter flow cytometric analysis was performed on PMBCs. The following immunophenotypic markers were used to define immune subsets: CD8^+^ T cells were CD4^−^CD8^+^, Tregs were CD8^−^CD4^+^CD25^high^FOXP3^+^. Cells were incubated with Fc receptor blocking agent and dead cells were excluded from all analyses by the means of LIVE/DEAD stain. The 34 495 CD8^+^ T cells (C1D1pre) and 23 948 CD8^+^ T cells (C2D1pre) were acquired and mean fluorescence intensity (MFI) for Ki67 and PD‐1 expressions were calculated. The 987 Treg cells (C1D1pre) and 564 Treg cells (C2D1pre) were acquired and MFI for Ki67 and CTLA‐4 expressions were calculated. (**a**) Change of Ki67 and PD‐1 expressions in CD8^+^ T cells after one cycle of atezolizumab plus chemotherapy in a patient with advanced carcinoid. PD‐1 and Ki67 in CD8^+^ T cells increased after one cycle of therapy. (**b**) Change of Ki67 and CTLA‐4 expressions in Tregs after one cycle of atezolizumab plus chemotherapy in a patient with advanced carcinoid. Ki67 and CTLA‐4 in Tregs increased after one cycle of therapy. Data were analyzed using FlowJo software (FlowJo LLC, OR, USA). Detailed methods are described in the Supplementary Text S1. (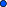) Ki67, (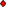) PD‐1, (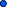) Ki67, (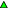) CTLA‐4

We evaluated a systemic immune response in the peripheral blood. Multiparameter flow cytometric analysis was performed on the peripheral blood mononuclear cells (PBMCs) as previously described (Supplementary Text S1).^13^, ^14^, ^15^ PD‐1 and Ki67 in CD8^+^ T cells increased after one‐cycle of chemoimmunotherapy, suggesting systemic immune activation was induced by blocking PD‐1/PD‐L1 pathway (Fig 3). However, CTLA‐4 and Ki67 expressions in Tregs simultaneously increased, indicating Treg activation was induced by blocking the PD‐1/PD‐L1 pathway.

## Discussion

Jiménez‐Sánchez *et al*. reported that multiple distinct TIME co‐exist within a single individual and heterogeneous TIME was associated with the heterogeneous fates of metastatic lesions,^8^ which were consistent with our findings. In the current study, a significant CD8^+^ T cell infiltration was seen in the tumor nest of the PD‐L1‐positive lung carcinoid, which was associated with a significant response to chemoimmunotherapy, despite Treg infiltration in both the tumor nest and stroma. However, CD8^+^ T cell infiltration was restrained in the surrounding stroma in the PD‐L1‐negative metastatic scapular tumor and which was associated with primary resistance to chemoimmunotherapy, suggesting that the infiltrating CD8^+^ T cells in the tumor nest may play a key role in response to ICIs in advanced carcinoid tumors.

Tregs have immunosuppressive activity and have been reported to play a critical role in negatively regulating antitumor immune responses.^16^ Activation of systemic Tregs with an increase of CTLA‐4 expression was confirmed after treatment with ICI followed by disease progression, suggesting that the CTLA‐4 signaling pathway could be one of the key mechanisms of acquired resistance to PD‐1/PD‐L1 blockade therapy in carcinoid.

Lung carcinoid tumors are categorized as typical (<2 mitoses per 2 mm^2^ and no necrosis) and atypical (2–10 mitoses per 2 mm^2^ and/or necrosis), corresponding to low‐grade (grade 1) and intermediate‐grade (grade 2), respectively.^17^ These categories were developed for resected primary tumors. A biopsied specimen represents only part of a whole tumor. Tumor heterogeneity within neuroendocrine tumors has been previously reported.^17^ Thus, the tumor heterogeneity of tissue specimens obtained by CT‐guided biopsies may have affected the difference of diagnosis in the current study.

Everolimus is an oral mammalian target of rapamycin inhibitor approved for NETs based on a clinical trial (RADIANT‐4).^18^ In this study, efficacy of everolimus compared to placebo in previously‐treated patients was reported. The current patient showed rapid progression of tumors at diagnosis. Therefore, we chose atezolizumab in combination with chemotherapy, which is currently used for poorly differentiated NET, small cell lung cancer as the first‐line therapy.

In our study, the PD‐L1‐positive primary tumor responded to therapy; however, the predictive value of PD‐L1 has not yet been validated in NET immunotherapy. Therefore, our findings should be interpreted with caution. Only one patient was involved in this study, thus further studies are needed to determine whether the principles discovered here apply to other patients with carcinoid tumors. Despite such limitations, the present study provides the first evidence of the coexistence of heterogeneous TIME within a single individual with a pulmonary carcinoid tumor. The present study may therefore contribute to our understanding of the TIME of carcinoids and provide new insights into the complex TIME of NETs.

## Disclosure

No potential conflicts of interest were disclosed.

## Supporting information


**Appendix** **S1**. Supporting Information
