# Supplementary material for: Heterogeneous tumor‐immune microenvironments between primary and metastatic carcinoid tumors differentially respond to anti‐PD‐L1 antibody therapy
Source: Thorac Cancer. 2020 Dec 9;12(3):397–401. doi: 10.1111/1759-7714.13772 (PMC7862786; doi:10.1111/1759-7714.13772)
Supplement: Supplementary file 1 — Appendix S1. Supporting Information [file TCA-12-397-s001.docx]

**Article type:** *Case Report*

**Heterogeneous tumor-immune microenvironments between metastatic carcinoid tumors differentially responding to anti-PD-L1 antibody therapy plus chemotherapy**

Shinya Sakata^1^, M.D., Ph.D., Kosuke Imamura^1^, M.D., Yuka Tajima^1^, M.D., Yuiko Masuda^1^, M.D., Ryo Sato^2^, M.D., Ph.D., Chieko Yoshida^1^, M.D., Ph.D., Shinichiro Okamoto^1^, M.D., Ph.D., Sho Saeki^1^, M.D., Yusuke Tomita^1^*****, M.D., Ph.D.**,** Takuro Sakagami^1^, M.D., Ph.D.

^1^Department of Respiratory Medicine, Kumamoto University Hospital, 1-1-1 Honjo, Chuo-ku, Kumamoto 860-8556, Japan

^2^Laboratory of Stem Cell and Neuro-Vascular Biology, Genetics and Developmental Biology Center, National Heart, Lung, and Blood Institute, National Institutes of Health, United States.

***Corresponding author**: Yusuke Tomita M.D., Ph.D., Department of Respiratory Medicine, Graduate School of Medical Sciences, Kumamoto University, 1-1-1 Honjo, Chuo-ku, Kumamoto, 860-8556, Japan. Tel: +81-96-373-5012. Fax: +81-96-373-5328. E-mail: [y-tomita@kumadai.jp](mailto:y-tomita@kumadai.jp), ORCID: <http://orcid.org/0000-0002-9680-7559>

**Supplementary Materials and Methods**

**Fluorescent multiplex immunohistochemistry**

Fluorescent multiplex immunohistochemistry was performed with OPAL Multiplex Fluorescent Immunohistochemistry Reagents (PerkinElmer, Waltham, MA, USA) following the manufacturer’s protocol and as described previously ^1^. Formalin-fixed paraffin-embedded (FFPE) sections of lung and scapular tumors were stained by one of the two sequences of primary antibodies, PD-L1 (clone E1L3N, rabbit, Cell Signaling, MA, USA), Synaptophysin (clone YE269, rabbit, abcam, Cambridge, United Kingdom) and CD8 (clone C8/144B, mouse, Nichirei, Tokyo, Japan), or Synaptophysin, FOXP3 (clone 236A/E7, mouse, abcam, Cambridge, United Kingdom), and CD3 (clone SP7, rabbit, abcam, Cambridge, United Kingdom), respectively. Nuclei were counterstained with 40,6-diamidino-2-phenylindole dihydrochloride (DAPI) (DOJINDO, Kumamoto, Japan). Multiplex slides were observed with a fluorescence microscope (BZ-X700, Keyence, Osaka, Japan). Scale bars (50µm) are shown in each panel. The inserted panel in right panel shows a CD3^+^FOXP3^+^ T-cell (Treg) at high magnification. High-speed scanning of whole slide images was performed on stained tissue sections. Images of full tissue sections were acquired with BZ-X700 using a 20x objective. For comparison of quantitative marker expression, images were analyzed with StrataQuest (TissueGnostics, Vienna, Austria). For nuclei and tumor detection, size and staining intensity of DAPI and synaptophysin were adjusted. Then, cutoff thresholds of CD8, CD3 and FOXP3 were determined by two independent observers. Finally, all images and generated statistics including number of CD8 positive cells and both CD3 and FOXP3 positive cells (Tregs) were automatically analyzed by StrataQuest. If counted cells localized inside the area of synaptophysin positive cells, those cells were counted as inside (tumor nests), if not, outside (tumor stroma).

**Peripheral blood immune subset analyses**

Blood samples were collected before therapy on cycle 1 day 1 (C1D1pre), and prior to treatment on C2D1 (C2D1pre) in cell preparation tubes with sodium citrate (BD Vacutainer CPT Tubes, BD Biosciences, NJ, USA). Peripheral blood mononuclear cells (PBMCs) were obtained by centrifugation and viably frozen until analysis. Multiparameter flow cytometric analysis was performed on PBMCs as described previously ^2-5^. Briefly, cells were incubated with Fc receptor blocking agent (Miltenyi Biotec, Bergisch Gladbach, Germany) and stained for 20 min at 4°C in a dark room with monoclonal antibodies. The following immunophenotypic markers were used to define immune subsets: CD8^+^ T-cells were CD4^−^CD8^+^; Tregs were CD8^−^CD4^+^CD25^hi^Foxp3^+^. The following monoclonal antibodies were used: PE-CD8 clone BNI3, APC/Cy7-CD4 OKT4, PerCP/Cy5.5-CD25 clone BC96, Alexa Fluor 488-Foxp3 clone 150D, Brilliant Violet 421-PD-1 clone EH12.2H7, PE/Cy7-CTLA-4 clone BNI3, and APC-Ki-67 clone Ki-67 (all from BioLegend, CA, USA) were used for T-cells. For analysis of Foxp3 expression, cells were fixed and permeabilized using a Fix/Perm buffer (Thermo Fisher SCIENTIFIC, MA, USA) according to the manufacturer’s instructions, then labeled with anti-Foxp3 antibody. Matched isotype controls were used for each antibody to determine the gates. Live cells were discriminated by means of LIVE/DEAD Fixable Aqua Dead Cell Stain (Thermo Fisher Scientific, MA, USA) and dead cells were excluded from all analyses. All flow cytometric analyses were performed using a BD FACSVerse™ (BD, Franklin Lakes, NJ, USA). Flow cytometric data for distinct parameters were quantified as the mean fluorescence intensity (MFI), as indicated. Data were analyzed using FlowJo software (FlowJo LLC, OR, USA).

**REFERENCES**

1. Saruwatari K, Sato R, Nakane S, et al. The Risks and Benefits of Immune Checkpoint Blockade in Anti-AChR Antibody-Seropositive Non-Small Cell Lung Cancer Patients. *Cancers (Basel)* 2019;11.

2. Sato R, Imamura K, Sakata S, et al. Disorder of Coagulation-Fibrinolysis System: An Emerging Toxicity of Anti-PD-1/PD-L1 Monoclonal Antibodies. *J Clin Med* 2019;8.

3. Apolo AB, Nadal R, Tomita Y, et al. Cabozantinib in patients with platinum-refractory metastatic urothelial carcinoma: an open-label, single-centre, phase 2 trial. *Lancet Oncol* 2020;21:1099-1109.

4. Thomas A, Rajan A, Berman A, et al. Sunitinib in patients with chemotherapy-refractory thymoma and thymic carcinoma: an open-label phase 2 trial. *Lancet Oncol* 2015;16:177-186.

5. Tomita Y, Lee MJ, Lee S, et al. The interplay of epigenetic therapy and immunity in locally recurrent or metastatic estrogen receptor-positive breast cancer: Correlative analysis of ENCORE 301, a randomized, placebo-controlled phase II trial of exemestane with or without entinostat. *Oncoimmunology* 2016;5:e1219008.
